# Stereotyped CLL-like immunoglobulin rearrangements in healthy repertoires: implications for leukemogenesis

**DOI:** 10.3389/fimmu.2026.1949983

**Published:** 2026-09-30

**Authors:** Davide Bagnara, Monica Colombo, Fabio Ghiotto, Niccolò Cardente, Giovanna Cutrona, Manlio Ferrarini, Franco Fais

**Affiliations:** 1 Department of Experimental Medicine, University of Genoa, Genoa, Italy; 2 Molecular Pathology Unit, AOM - IRCCS Ospedale Policlinico San Martino, Genova, Italy; 3 Department of Immunology, University of Oslo, Oslo, Norway

**Keywords:** B lymphocytes, B-cell receptor (BCR), chronic lymphocytic leukemia (CLL), IGHV repertoire, stereotyped immunoglobulin

## Abstract

Chronic lymphocytic leukemia (CLL) is characterized by a highly biased B-cell receptor (BcR) immunoglobulin repertoire and by recurrent stereotyped receptors defined as quasi-identical BcR recurring in unrelated patients. High-throughput sequencing studies have shown that immunoglobulin rearrangements resembling these CLL stereotypes can also be detected in healthy individuals. Beyond their relevance to leukemogenesis, these findings provide a distinctive opportunity to investigate how recurrent BcR configurations are generated, selected, and maintained during normal B-cell development. In this review, we examine the occurrence of CLL-like stereotyped immunoglobulin rearrangements in healthy repertoires and discuss their implications for normal B-cell biology. Available studies indicate that these rearrangements are physiological components of the repertoire, detectable in cord blood, bone marrow, spleen, and peripheral blood, and distributed across transitional, naïve, marginal zone-like, and memory B-cell compartments. Their representation is non-random and subset specific, suggesting that different receptor configurations are subject to distinct developmental, tolerance, and antigen-driven selection pressures. Some rearrangements are preferentially associated with antigen-inexperienced compartments, whereas others are more frequently found in post-germinal-center populations, partially recapitulating the mutational context of their leukemic counterparts. However, healthy B cells generally lack the stringent light-chain restriction observed in established CLL subsets, indicating that leukemic stereotypy results from additional receptor refinement and clonal selection. Overall, the study of CLL-like stereotypes in healthy individuals extends beyond the identification of putative leukemic precursors. It reveals how convergent receptor architectures arise within physiological immunity, how autoreactive or polyreactive configurations may escape complete censoring, and how normal B-cell repertoires accommodate recurrent immunogenetic solutions without necessarily progressing to malignancy.

## Introduction

Chronic lymphocytic leukemia (CLL) is regarded as an antigen-driven disease in which the B-cell receptor immunoglobulin (BcR) plays a central biological role, as supported by the biased immunoglobulin repertoire of CLL, where specific *IGHV*, *IGH*D and *IGHJ* gene segments and combinations thereof are overrepresented and (1, 2) by the presence of stereotyped BcR IG, which are quasi-identical receptors, recurring in unrelated patients, sharing highly similar HCDR3 motifs together with restricted heavy- and light-chain gene usage (3–7). Such recurrence is unlikely to be stochastic and indicates that common selective pressures, most plausibly antigenic, have shaped leukemic clonal expansion. These immunogenetic features are also clinically relevant. *IGHV* mutational status is a strong prognostic marker in CLL, separating cases with aggressive behavior (unmutated, U-CLL) from those with indolent course (mutated, M-CLL) (8, 9). In addition, BcR stereotypy further refines disease heterogeneity. Stereotypes are defined into distinct subsets (4) and the expression of BcR of a given subset associates with distinct cellular features, signaling properties and clinical behavior of single cases (10–14). Thus, the leukemic BcR represents a determinant of disease biology and a key therapeutic vulnerability, as illustrated by the efficacy of inhibitors of BTK (a major component of the BcR signaling pathway) in CLL (15).

Within this framework, the identification of CLL-like stereotyped rearrangements in healthy individuals is particularly informative. If receptors resembling CLL stereotypes are already detectable in normal B-cell repertoires, then part of the immunogenetic space exploited in leukemia must pre-exist within physiological immunity. Studying these healthy counterparts may help clarify how often such receptors are generated during normal repertoire formation, whether they are preferentially expressed in specific B-cell compartments, and whether different stereotyped subsets follow distinct physiological maturation routes before malignant transformation, collectively providing clues about both the developmental trajectory and the cellular context from which the different CLL clones emerge. The presence of a CLL-like rearrangement in healthy donors does not imply that the cell bearing it is a leukemic precursor. Rather, comparison between healthy and leukemic repertoires and the features of CLL-like stereotypes in normal and full blown CLL may help distinguish BcRs which belong to the normal or convergent repertoire from those undergoing additional selection during leukemogenesis. This may explain why certain stereotyped subsets are relatively frequent in health but uncommon in CLL, whereas others show the opposite pattern. Ultimately, the study of CLL-like stereotypes in healthy repertoires is relevant because it may reveal the earliest steps that precede overt disease, while also highlighting the selective bottlenecks that transform a physiologically generated receptor into a disease-defining BcR.

Early pre-NGS studies provided evidence that immunoglobulin rearrangements with similarities to CLL stereotyped B-cell receptors exist in normal B cells (16–18). However, these studies did not provide a comprehensive assessment of repertoire-wide prevalence or distribution because they were low-throughput, locus-restricted or focused on selected B-cell compartments. For this reason, the present review focuses primarily on NGS-based studies, which enabled broader and more systematic analysis of CLL-like stereotyped rearrangements across healthy donors, tissues and B-cell subpopulations (see **Table 1** for a summary).

**Table 1:** NGS / high-throughput repertoire studies analyzed in the review.

Study	Biological material / cohort	Technology / scope	How stereotypy was operationalized	Main points	Frequency range of identified total stereotyped rearrangements
Muggen et al., 2019 (28)	Peripheral blood B-cell subsets from 155 healthy individuals aged 20–95 years	NGS + multiparameter flow cytometry	Healthy sequences assigned against a reference database of stereotypic CLL BCRs based on HCDR3 characteristics;	Reports that CLL-associated stereotypic BCRs are present in healthy repertoires and that some subsets increase with age	0.17-0.62%
Colombo et al., 2020 (30)	Splenic B-cell subsets plus CD5+ and CD5− B cells from spleen and peripheral blood	High-throughput immunogenetic approach / NGS, but restricted to IGHV1-bearing rearrangements	Search for CLL-biased stereotyped immunoglobulins (CBS-IG); distinguishes typical vs non-typical CBS-IG within an IGHV1-centered design	Shows that CLL-biased stereotypes can be detected across multiple normal B-cell subsets, with enrichment in CD5+ compartments	0.17-0.57%
Agathangelidis et al., 2021 (31)	Individuals with low-count MBL plus age-matched healthy donors (naive and memory B cells)	High-throughput repertoire analysis	Purpose-built clustering algorithm; assessed abundant and expanded clonotypes and searched for major CLL stereotyped clonotypes	Important transitional study linking healthy normal B cells, LC-MBL and overt CLL; major CLL-specific stereotypes were infrequent and low-abundance	0.0002-0.28%
Bagnara et al., 2022 (26)	Circulating B-cell subpopulations from 9 healthy donors, also fractionated by CD5 and light chain	High-throughput sequencing + ARResT/AssignSubsets after pre-filtering	ARResT-based frameworks in healthy donors; defines CLS-IG and explicitly distinguishes typical vs non-typical CLS-IG	Strong methodological paper for comparing distribution of CLS-IG across subsets, CD5 status and light-chain usage	0.35-0.10
Vergani et al., 2023 (29)	Cord blood, bone marrow and peripheral blood from healthy donors; also single-cell heavy/light chain data	Bulk repertoire sequencing plus single-cell sequencing	Custom but explicit Stamatopoulos-group clustering rules for standard CLL-SLS and satellite CLL-SLS	Shows that CLL stereotype-like sequences are detectable across developmental stages and are not obviously censored by central/early peripheral tolerance	0.2-0.80%
Dupont et al., 2025 (27)	Bulk circulating BCR repertoires from 69 healthy volunteers plus a longitudinal public dataset of sorted B-cell subpopulations	High-throughput repertoire analysis on a larger healthy-volunteer series	ARResT-oriented major subset framework; explicitly analyzes both typical and non-typical CLS-IG as clonotypic families	Provides the clearest large-cohort healthy-volunteer estimate of prevalence and abundance; supports the view that healthy CLS-IG are largely public and mostly pre-GC	0.053%

BCR, B-cell receptor; CLS-IG, CLL-like stereotyped immunoglobulin; CBS-IG, CLL-biased stereotyped immunoglobulin; CLL-SLS, CLL stereotype-like sequence; LC-MBL, low-count monoclonal B-cell lymphocytosis; GC, germinal center; pre-GC, pre-germinal center.

## CLL BcR stereotypy and the concept of public antibody sequences

The term stereotyped BcR refers to non-randomly recurring IG receptors which share highly constrained immunogenetic features, including the use of phylogenetically related *IGHV* genes and closely similar HCDR3 length, composition and motif architecture.

The conceptual roots of this phenomenon predate modern high-throughput repertoire analysis. Earlier work on shared or public idiotypes already suggested that unrelated individuals can generate antibodies with recurrent, non-random variable-region features (reviewed in (19)). In this sense, the modern sequence-based concept of a public clonotype can be viewed as a reformulation of an older idea of interindividual antibody convergence, whereas CLL stereotypy represents a disease-oriented extension of that broad principle. However, stereotypy cannot be equated with the stricter concept of public clonotypes which, like stereotypes, are shared by different individuals, but use the same V and J genes and an identical HCDR3 amino acid sequence (20, 21) reflecting recurrence at near-identity level.

CLL BcR stereotypy was developed as a disease-oriented framework to capture a broader form of immunogenetic convergence. As summarized by Darzentas and Stamatopoulos (22), the criteria evolved from early approaches requiring shared *IGHV/IGHD/IGHJ* genes between different patients, identical *IGHD* reading frame and high HCDR3 identity, to later rule-based definitions grouping receptors with quasi-identical antigen-binding sites. In its mature form, this framework incorporated criteria such as at least 50% HCDR3 amino acid identity, at least 70% similarity, usage of *IGHV* genes from the same phylogenetic clan, identical HCDR3 length, and identical motif offset. Thus, stereotypy in CLL is not based on clonotype identity, but on shared structural and immunogenetic constraints interpreted as evidence of convergent selection.

Based on the above considerations, CLL stereotypes which are detected in healthy individuals are more appropriately described as CLL-like stereotyped immunoglobulin gene rearrangements (CLS-IG) rather than public clonotypes. Their designation reflects similarity rather than identity to CLL-defined stereotyped receptors and does not provide formal proof of a precursor-product relationship between normal and leukemic cells. Thus, CLS-IG represent convergent immunogenetic solutions conceptually overlapping with the public repertoire, without fulfilling the public clonotype definition.

The public repertoire literature lends support to the above considerations. Briney et al. (20) showed that even an exceptionally diverse baseline antibody repertoire contains a measurable fraction of clonotypes shared across individuals, while Soto et al. (21) demonstrated that shared clonotypes are already detectable in cord blood and can persist into adulthood. Together, these observations indicate that recurrent receptor configurations can arise physiologically, before or independently of overt antigen-driven clonal expansion. For the CLL field, this means that recurrence of CLL-like stereotyped rearrangements in healthy donors is not, by itself, evidence of leukemic potential, but rather evidence that the normal repertoire can generate convergent receptor architectures later exploited and further selected during CLL evolution.

A further note is that, although CLL stereotypy is primarily defined based on heavy-chain features, the concept is effectively reinforced by recurrent and subset-specific light-chain usage (5, 23–25) in several major subsets. This is well established not only for subset #2, but also for subsets #1, #4, #6, #8, #64B and #99 (see **Table 2**). At present, however, it remains unclear whether an analogous level of paired heavy- and light-chain conservation exists in the normal repertoire, since no large-scale data are available showing public BcR defined by combined heavy- and light-chain sequence identity or convergence.

**Table 2:** Indication of which major CLL subsets are most commonly retrieved, selectively observed, or typically underrepresented in healthy immune repertoires across the studies reviewed.

CLL subset, heavy chain, mutational status, IGHV gene(s) or family(ies) associated in CLL clones	HCDR3 aa length	Pattern in healthy repertoire	Main supporting observations across studies	Overall interpretation
#2 (IGM, M and U) IGHV3-21	9	Recurrently identified	Prominent in naive mature B cells in Muggen et al.; among the more represented CLS-IGs in Bagnara et al.; highly prevalent in healthy volunteers in Dupont et al.; recurrent in multi-tissue analysis by Vergani et al.	One of the most reproducible CLL-like stereotypes in health.
#14 (*, M) IGHV4	10	Recurrently identified	Particularly evident in antigen-experienced compartments in Muggen et al.; reproducibly detected in mature B-cell subsets in Bagnara et al.; among the most represented standard CLL-SLS in Vergani et al., including cord blood and bone marrow.	One of the most consistently recovered mutated / antigen-experienced-associated stereotypes in health.
#5 (IGM, U) IGHV1-69	20	Often identified	Among the most represented CLS-IGs in Bagnara et al.; prominent in naive mature B cells in Muggen et al.; recurrent in Dupont et al.	Common in healthy repertoires, though less uniformly emphasized than #2 and #14.
#64B (IGM, U) IGHV 3 (Clan III)	21	Often identified	Recurrent in naive mature B cells in Muggen et al.; among the most represented CLS-IGs in Bagnara.	Likely part of the recurrent healthy CLL-like space, especially in peripheral blood studies.
#28A (IGM, U) IGHV1-2	17	Prominent in selected studies	Strongly represented in the IGHV1-focused study by Colombo et al.; recurrent in Vergani bone marrow / peripheral blood datasets.	Relevant but more study-dependent and influenced by analytical design.
#73 (**, M) IGHV3-30	12	Prominent in selected studies (Vergani et al)	Recurrent in Vergani et al., especially in cord blood and peripheral blood.	Important in the multi-tissue study, but not yet a broadly replicated cross-paper signal like #2 or #14.
#148B (**, M) IGHV2-5	17	Prominent in selected studies (Vergani et al)	Highly represented (Vergani et al.) in bone marrow / peripheral blood analyses.	Appears recurrent in some tissues, but not consistently emphasized across all studies.
#1/#99 (IGM, U) IGHV 1/5/7 (clan I)	13/14	Usually underrepresented / sometimes absent	Low frequency in Bagnara et al.; absent as a typical clonotype in Dupont et al. despite being a major CLL subset.	Strong example of discordance between health and leukemia.
#4 (IGG, M) IGHV4-34	20	Usually rare or absent	Rare or absent in Bagnara et al.; also not among the dominant healthy-donor subsets in the other studies. Reported in Agathangelidis et al., in the unmutated repertoire.	One of the clearest examples of a subset prominent in CLL but poorly represented in health.
#8 (IGG, U) IGHV4-39	19	Usually rare or absent	Rare or absent in Bagnara et al.; not among recurrent healthy-donor subsets in the other studies.	Similar to #4: uncommon in healthy repertoire despite its importance in CLL.

The first and second columns describe the immunogenetic features of each subset in the CLL context.

CLS-IG, CLL-like stereotyped immunoglobulin; CLL-SLS, CLL stereotype-like sequence.

*For this subset, cases expressing both IgM and IgG were identified in our cohorts;

**For this subset, heavy-chain assignment was not possible, as the subset was absent in our cohorts.

## Operational definitions of CLL-like stereotypy in healthy repertoires

Differences in “stereotypy” definition are probably one of the main reasons of the discrepancies in the reported frequencies of CLL-like stereotyped rearrangements in healthy donors. Although based on the same general concept that B-cell receptors from unrelated subjects can show non-random similarity in HCDR3 structure together with similar *IGHV* usage, the methodologies differ in how strictly these similarities are measured and in how broadly the search is performed.

In certain studies (26–28), stereotypy was defined through ARResT/AssignSubsets (https://bat.infspire.org/arrest/assignsubsets/), which can be regarded as a standardized and relatively rigid implementation of the broader Stamatopoulos/Agathangelidis immunogenetic framework for major subset assignment. This framework operationalizes assignment to the original 19 so-called major CLL subsets defined in the field (4). The study of Bagnara et al. (26) provides an example of this way of operating. Sequences were first preselected according to the core features of the 19 major CLL stereotyped subsets, *IGHV* clan, *IGHV* mutational status, and HCDR3 length, and then submitted to ARResT/AssignSubsets, and only those sequences reaching at least average confidence were retained as CLS-IGs. Because this approach is explicit and tool-based, the results are easier to compare across cohorts.

Other studies used more customized approaches (29–31). Vergani et al. applied rules derived from the Stamatopoulos-group framework and included more permissive “satellite” sequences, thereby widening the search space. A similar approach was utilized by Agathangelidis et al. in their studies on low-count MBL and healthy donors. These broader approaches may detect additional rare or borderline stereotype-like rearrangements, including subset #4-like sequences, that were not retrieved in the more strictly ARResT-based studies. These results do not indicate a capacity of detecting more stereotype-like sequences and more likely reflect a broader operational stereotype definition.

A separate case is represented by the study of Colombo et al. (30), where the analysis was intentionally restricted to *IGHV1*-bearing rearrangements. This was therefore a more selective search and should not be directly compared with whole-repertoire studies when considering the recoverability of other stereotyped subsets.

Overall, the key distinction is not simply between “stringent” and “non-stringent” methods, but between standardized tool-based approaches and study-specific customized approaches that differ in how broad the search for related sequences is and prevent comparisons across studies. For this reason, frequencies of CLL-like stereotyped rearrangements should not be compared across studies without considering how the search was performed. It should also be noted that, across the studies considered, the frequency of the so-called major subsets in normal repertoires is relatively variable, likely reflecting both biological and methodological differences, but remains consistently low and, where directly measurable, never exceeds 0.8% of total rearrangements (details are provided in **Table 1**).

## Distribution across tissues, B-cell compartments and age groups

CLS-IG have been described in peripheral blood, spleen, bone marrow and cord blood (see **Tables 1**, **2** for details), indicating that they are not confined to circulating mature B cells. At the same time, the composition of the CLS-IG space differs by tissue. Cord blood may already contain CLS-IG (29), but with a narrower subset distribution than adult bone marrow or peripheral blood. Likewise, splenic (30) and peripheral blood repertoires contain CLL-like rearrangements, but the relative representation of individual subsets does not reproduce that observed in CLL. Thus, tissue context influences the representation of specific stereotyped configurations even when the broader CLL-like space is detectable across compartments.

CLS-IG have been identified by NGS across transitional, naïve, marginal zone-like and multiple memory cells, and in some studies also in earlier B cell developmental stages in bone marrow. However, their distribution is not uniform. Some reports suggest enrichment of CLS-IG rearrangements in antigen-inexperienced or pre-germinal-center compartments, particularly transitional and naïve B cells. This is not unexpected, since these subpopulations are enriched in *IGHV*-unmutated B cells and most CLS-IG are likewise unmutated. However, selected stereotypes may also appear in antigen-experienced populations (see **Table 2** for details).

In this context, the observations on normal CD5+ B cells deserve particular consideration, as they have contributed to the debate on the cellular origin of CLL. Seifert et al. (18) reported an enrichment of CLL-like stereotyped IG rearrangements in mature CD5+ B cells and, importantly, distinguished an unmutated CD5+CD27− population from a smaller, somatically mutated CD5+CD27+ post-GC population, proposing these as possible normal counterparts of U-CLL and M-CLL, respectively. Thus, their findings cannot be explained solely by the higher proportion of unmutated B cells within the bulk CD5+ compartment.

Subsequent NGS-based studies, however, have not confirmed that CD5+ B cells represent a uniquely or preferentially enriched normal counterpart of CLL. In Bagnara et al. (26), the higher representation of unmutated CLS-IG within bulk CD5+ B cells could at least partly reflect the enrichment of unmutated cells in this compartment, but this observation does not directly address the mutated CD5+CD27+ population identified by Seifert et al. Moreover, direct comparison between these studies should be made cautiously, because they differed substantially in sequencing depth and in the operational criteria used to assign stereotypy, with earlier studies applying broader similarity-based definitions than the more standardized subset-assignment approaches used subsequently.

Taken together, the available data support the presence of CLL-like stereotyped rearrangements within normal CD5+ B-cell populations, including both pre- and post-GC compartments, but do not establish CD5 expression as evidence of a unique or direct lineage relationship with CLL. Indeed, the healthy-repertoire studies analyzed in this review, including those examining purified B-cell subpopulations across different developmental compartments, identified CLS-IG in several subsets without revealing a single population sufficiently enriched to support a unique normal counterpart for the leukemic clone.

The relationship with age is less fully concordant across studies. Muggen et al. (28) reported an increase in selected CLL-associated stereotypic rearrangements mainly in individuals older than 70 years, whereas no comparable signal was evident in the intermediate 50–70-year groups. By contrast, Dupont et al. (27), who defined older donors more broadly, including individuals aged 55–78 years in a single category did not find such increase with age. This difference in age stratification likely explains at least part of the apparent discrepancy: if the enrichment is largely driven by the oldest subjects, pooling donors aged 55–69 with those over 70 would dilute the signal.

Overall, the available evidence supports a model in which CLS-IG are physiological components of the normal repertoire, distributed across tissues and differentiation stages, but shaped by compartment-specific and age-related selection pressures. At the same time, these studies have not identified a single normal B-cell subpopulation specifically enriched in CLL-like stereotyped rearrangements to an extent that would support a direct lineage relationship with the leukemic clone. This point is relevant because earlier models had proposed a more specific derivation of CLL from restricted normal B-cell compartments (32), including marginal zone-related populations. By contrast, the current immunogenetic data are more consistent with the possibility of multiple developmental routes, rather than a single normal counterpart for all CLL clones.

## Stereotype subset-specific patterns in healthy repertoires

The subset distribution of CLL-like stereotyped rearrangements in healthy individuals is non-random although it does not mirror the hierarchy of CLL, since some stereotyped subsets are reproducibly identified, whereas others, that are prominent in leukemia, are consistently rare or absent. Thus, the normal repertoire does not simply contain a diluted version of the leukemic stereotyped landscape but shows a distinct distribution of convergent rearrangements shaped by physiological repertoire generation and compartment-specific selection (**Figure 1**). Subset #2 stands out as one of the most represented. It is prominent in naïve mature B cells in Muggen et al. (28), recurrent across peripheral blood B-cell subsets in Bagnara et al. (26), and highly prevalent in the large healthy-volunteer series analyzed by Dupont et al. (27). A similarly recurrent pattern is seen for subset #14, which appears repeatedly in normal repertoires and is often more evident in antigen-experienced compartments. Other recurrent subsets include #5 and #64B in peripheral blood, and, in some tissue-based analyses, #28A, #73 and #148B.

**Figure 1:**
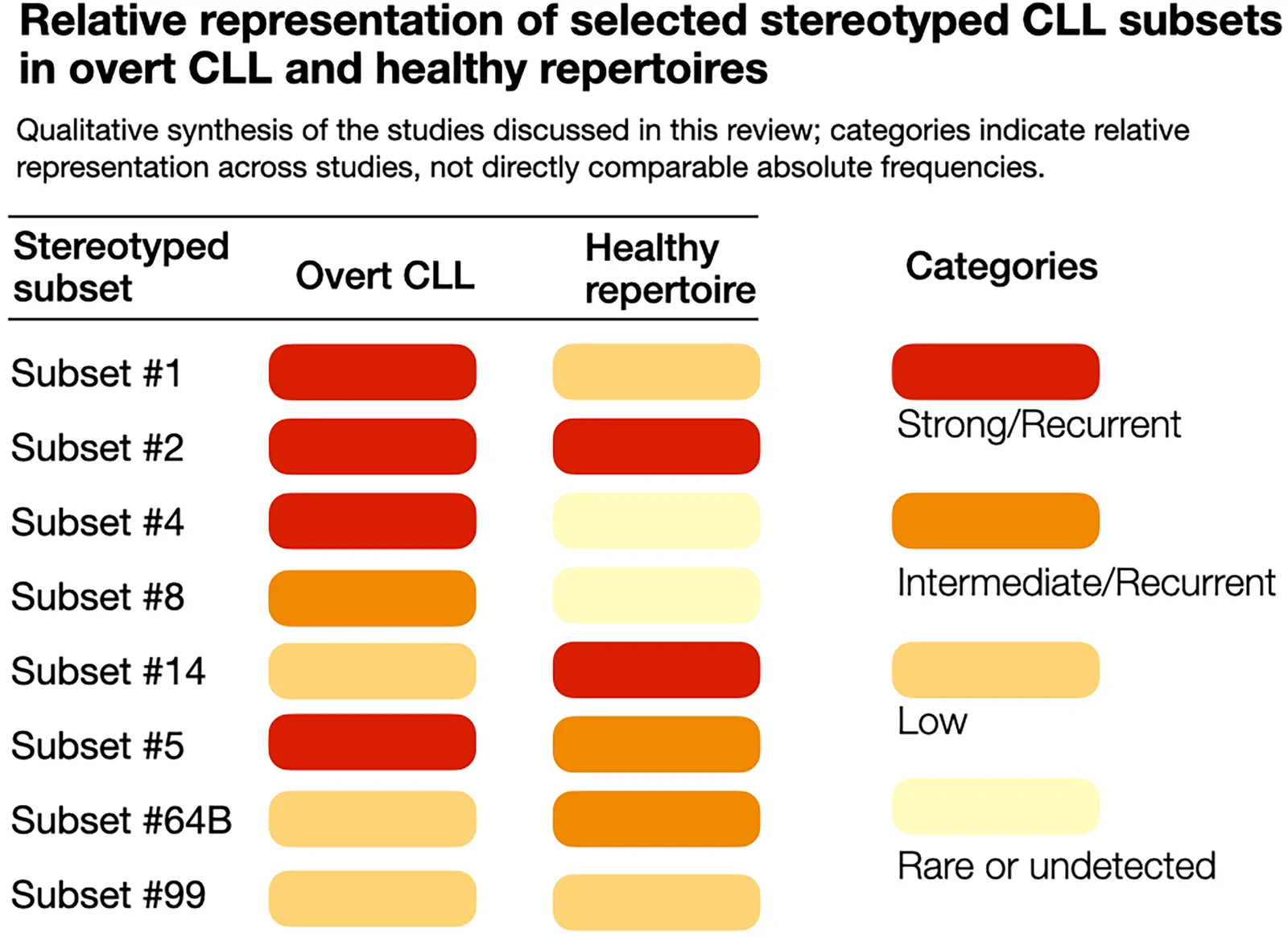
Relative representation of selected stereotyped CLL subsets in overt CLL and healthy repertoires. For healthy repertoires, categories summarize the consistency of subset recovery across the studies reviewed rather than directly comparable absolute frequencies: “strong/recurrent” indicates subsets repeatedly identified across ≥3 independent studies and consistently reported among the more represented CLS-IG; “intermediate/recurrent” indicates recurrent detection in at least two studies but with less consistent representation; “low” indicates subsets detected at generally low or inconsistent representation; and “rare or undetected” indicates subsets absent from most studies or only sporadically detected. Classification was based on the studies summarized in **Tables 1**, **2**. For overt CLL, categories were assigned according to the relative representation of the major stereotyped subsets reported by Agathangelidis et al. (Blood, 2012;119:4467–4475). The color scale should therefore be interpreted as a qualitative synthesis and not as a direct quantitative comparison between CLL and healthy repertoires.

By contrast, several subsets that are abundant in CLL are underrepresented in healthy repertoires. Subsets #4 and #8 are usually rare or undetectable in healthy repertoire, and #1 is often less represented despite its importance in CLL. Non-detection should not be equated with true absence, since only a fraction of everyone’s total B-cell repertoire is experimentally sampled, even in deep-sequencing studies and the apparent scarcity of certain subsets may reflect both biological rarity and incomplete repertoire coverage. Altogether, these findings support the idea that the distribution of stereotypes in CLL reflects not only the physiological generation of convergent BcR rearrangements, but also subset-specific selection during malignant evolution. This conclusion should nevertheless be interpreted with caution, since apparent recurrence is influenced not only by repertoire scope, tissue source, sequencing depth and operational definition, but also by technical variables related to preparation, amplification and analysis of the starting genetic material. Even so, comparative considerations remain possible, particularly for those subsets that show at least partially overlapping patterns across independent studies. Other factors may influence the probability of identifying individual CLL-like stereotypes in healthy repertoires. HCDR3 length may be relevant, since shorter HCDR3s are generally more compatible with interindividual recurrence (20, 21, 33). This may help explain why subsets like #2 and #14, with very short HCDR3s, are recurrently detected in healthy donors. However, several stereotypes with longer HCDR3, like #5 and #64B, also are frequently identified, whereas others remain rare, suggesting that HCDR3 length is not a sole or a major player. A more informative variable may be the relationship with the mutational status of the corresponding CLL subset. Indeed, CLL-like stereotyped rearrangements in healthy subjects may retain the *IGHV* mutational bias of their leukemic counterparts, with M-CLL-associated stereotypes being more frequent in antigen-experienced cells and U-CLL-associated stereotypes more numerous in antigen-inexperienced cells. Thus, the frequency of individual CLL-like stereotypes in health may reflect a combination of convergent generation, HCDR3 architecture and compatibility with the compartment-specific mutational context.

As noted above, in CLL the concept of stereotypy is not limited to heavy-chain features alone, but in several major subsets is reinforced by a highly restricted light-chain configuration, often involving recurrent usage of a specific *IGKV* or *IGLV* gene in combination with the subset-defining heavy chain. This aspect has been addressed only partially in healthy-donor studies. In Bagnara et al. (26), the analysis was performed on κ- versus λ-expressing B-cell subpopulations rather than on paired heavy/light-chain sequences. In this setting, no comparable light-chain restriction was observed for the informative CLS-IG subsets examined, including #1, #2 and #64B, despite the highly constrained light-chain gene usage characteristic of these subsets in CLL. Vergani et al. extended the analysis by combining κ/λ-based sorting with targeted single-cell paired IGH–IGL sequencing and showed a more heterogeneous picture. For #2/#169, which in CLL is associated with a highly recurrent *IGLV3-21*-containing receptor configuration, no comparable restriction was observed in normal B cells. Conversely, for some *IGHV4-34*-associated subsets, more subset-consistent heavy/light-chain pairings could be identified, including examples related to #201 and #4, although these still did not fully reproduce the complete leukemic receptor configuration (29). Thus, whereas recurrent heavy/light-chain pairing are robust features of several established CLL stereotypes, current healthy-repertoire data do not demonstrate a comparable degree of receptor-level restriction. This conclusion remains limited by the scarcity of large-scale paired heavy/light-chain repertoire studies in healthy individuals.

## Subset-specific observations and proposed CLS-IG trajectories

The BcR is a major determinant of CLL biology and may also influence the persistence or expansion of normal B cells carrying receptor configurations compatible with those observed in CLL. Such effects could involve different mechanisms, including auto-/polyreactivity (34–36), autonomous BcR-BcR interactions (37), and antigenic stimulation by pathogens (38, 39). Although these properties may coexist, they represent distinct biological processes and do not necessarily co-segregate across stereotyped subsets. Subset #1 provides an informative example, displaying prominent auto-/polyreactivity without detectable autonomous signaling (36).

It is important to distinguish between observations made directly in healthy B-cell repertoires, functional properties demonstrated for leukemic BcR, and developmental trajectories inferred by integrating these two types of evidence. The latter should therefore be regarded as working models rather than demonstrated precursor-product relationships.

Despite methodological differences, one relatively consistent observation from healthy-donor studies is that CLS-IG tend to be detected in B-cell compartments sharing the mutational features of the corresponding leukemic stereotypes. Thus, stereotypes that are usually unmutated in CLL are more frequently identified in antigen-inexperienced, naïve-like B cells, whereas stereotypes that are generally mutated in CLL are more often found in antigen-experienced or post-germinal-center populations. This correspondence is looser in studies using broader, personalized Stamatopoulos-derived approaches (29–31) and clearer in studies applying stricter ARResT-based criteria (26–28). These observations indicate developmental compatibility between healthy CLS-IG and their leukemic counterparts, but do not establish direct lineage continuity.

For instance, subset #1 is a prototypical unmutated CLL stereotype, characterized in leukemic cells by absence of somatic hypermutation, IgM expression and marked auto-/polyreactivity. However, subset #1 BcR does not appear to rely on cell-autonomous signaling (36). In healthy repertoires, subset #1-like rearrangements are also found almost exclusively in naïve/unmutated compartments. The direct observation from healthy donors is therefore that the same broad mutational context is preserved. Whether the autoreactive/polyreactive properties demonstrated for leukemic subset #1 are already functionally expressed in healthy subset #1-like B cells has not been formally established. A plausible interpretation is that such receptor architectures may be physiologically generated and tolerated in naïve B cells, whereas additional events are required for their persistence, expansion, and eventual leukemic selection.

An additional difference concerns light-chain usage. In CLL, subset #1 shows a characteristic restriction to an Igκ light chain, whereas healthy subset #1-like rearrangements can be associated with either light-chain isotype. This indicates that the healthy and leukemic receptor configurations are not equivalent. One possible, but still unproven, interpretation is that leukemic evolution may involve additional selection of particular heavy- and light-chain combinations. However, because extensive information on paired heavy- and light-chain repertoires is still lacking, it is currently not possible to define the selective processes that may favor association of a given heavy chain with a particular light chain during leukemic evolution. The rarely detected subset #99, considered a subset #1-related satellite (6), shows a similar distribution, being largely confined to the naïve/unmutated repertoire of normal donors.

Subset #14 shows an almost opposite pattern. In CLL it is characteristically mutated, and in healthy donors subset #14-like rearrangements are repeatedly detected in antigen-experienced or post-germinal-center B-cell compartments. This constitutes direct evidence that the normal repertoire can generate subset #14-compatible configurations within the same broad maturation context as their leukemic counterparts. However, it does not demonstrate that these cells represent immediate precursors of subset #14 CLL.

Subset #2 is particularly informative because the conventional mutated/unmutated dichotomy does not fully capture its biology. Although this subset includes both mutated and unmutated CLL cases, as defined by the conventional 2% cutoff, even the latter retain detectable somatic mutation, consistent with some degree of antigen-driven maturation. In healthy repertoires, subset #2-like rearrangements are relatively frequent and can be recovered across different B-cell compartments. What is directly supported by healthy-donor data is therefore the recurrent physiological generation and persistence of subset #2-compatible rearrangements. Their relatively high frequency may partly reflect the short HCDR3 and may also indicate that these receptor configurations are not strongly counter selected during normal B-cell maturation.

Functional studies in CLL provide an additional, but distinct, layer of information. In leukemic subset #2, acquisition of specific somatic changes contributes to self-recognition and autonomous signaling, including the somatically introduced arginine at the CL–VL junction (40). These findings suggest a possible developmental model in which the healthy repertoire contains multiple subset #2-compatible configurations, whereas only some undergo the additional structural refinement required for the full leukemic BcR phenotype. This interpretation is biologically plausible but has not been demonstrated longitudinally at the single-clone level.

Subset #4 provides an even clearer example of the distinction between observation and inference. In healthy-donor NGS studies, subset #4-like rearrangements are rare and have been detected particularly with broader, personalized assignment strategies. In CLL, by contrast, subset #4 is associated with *IGHV4-34*, IgG expression, somatic hypermutation and an indolent clinical course (5, 41). Germline-reverted subset #4 BcR from CLL cells display broader polyreactivity/autoreactivity, including reactivity against self and microbial antigens, whereas the mutated leukemic receptors show less overt autoreactivity, suggesting that SHM may have edited or attenuated the polyreactivity of their inferred precursors during or after germinal-center transit (reviewed in (42)). Clonal redemption provides a general physiological precedent for this type of process, whereby autoreactive B cells can enter germinal centers and acquire mutations that reduce self-reactivity (43).

These functional observations derive from leukemic or experimentally reconstructed receptors and should not be taken as direct evidence that healthy subset #4-like B cells follow the same sequence of events. Nevertheless, they suggest a possible trajectory distinct from that proposed for subset #1. Subset #4 CLL cells show biochemical and functional features consistent with anergy, including low surface IgG expression, constitutive ERK1/2 activation and poor responsiveness to BcR cross-linking (44). In addition, subset #4 BcR are capable of BcR-BcR interactions leading to autonomous signaling, which appears to depend on class-switch recombination to IgG because a critical residue involved in the conformational interaction is present in the IgG CH1 domain (40).

On the basis of these CLL-derived functional data, one may therefore hypothesize, rather than infer directly from healthy repertoires, a multistep trajectory for subset #4 involving: i) generation of an autoreactive/polyreactive *IGHV4-34*+ BcR configuration; ii) stimulation of B-cell descendants, potentially including superantigen-like interactions mediated by binding of polymeric N-acetyllactosamine-containing structures to the hydrophobic patch in the *IGHV4–34* FR1 region (45); iii) germinal-center-associated maturation with somatic hypermutation; and iv) class-switch recombination to IgG, which may enable the distinctive receptor interactions described in leukemic subset #4. This model is consistent with the highly mutated and IgG-switched phenotype of subset #4 CLL but remains largely inferential in the context of healthy repertoires, because genuine subset #4-like rearrangements have been only rarely observed outside CLL. Notably, the rare CLL subset #16, which shares *IGHV4–34* usage and IgG expression with subset #4, is also rarely detected in normal donors across studies.

Overall, the healthy-repertoire data do not identify single normal counterparts for individual CLL stereotyped subsets. Rather, they show that different CLS-IG are generated and maintained within distinct physiological B-cell compartments. Functional studies of the corresponding leukemic BcR suggest possible subset-specific mechanisms of selection, including autoreactivity, antigen dependence, somatic refinement and autonomous signaling. The developmental trajectories proposed here therefore represent models that integrate these two levels of evidence, rather than demonstrated pathways linking individual healthy CLS-IG-bearing cells to future leukemic clones. In this sense, stereotyped subsets provide useful examples of how physiological B-cell repertoire formation may intersect with, but should not be equated to, leukemic evolution.

## Concluding remarks and open questions

The presence of CLS-IG rearrangements in healthy donors indicates that part of the immunogenetic space later exploited in CLL already exists within normal immunity. At the same time, the detection of CLS-IG in normal B cells does not imply that they must evolve into leukemic clones. Rather, the comparison between healthy and leukemic repertoires indicates that many CLS-IG configurations may be generated physiologically, and that a minority of them undergo the additional selection required for leukemic evolution.

The detection, in apparently healthy individuals who later developed CLL, of clonotypes corresponding to the future leukemic clone, including stereotyped BcR IG in some cases (46) is different from the finding of CLS-IG in virtually all individuals most of whom will never develop pathology. The former observation just provides the detection of future CLL clonotypes in longitudinal samples from individuals who late developed CLL whereas the latter one indicates a repertoire feature with unknown evolutionary potential.

Different stereotyped subsets may arise from related auto-/polyreactive B-cell precursors through different evolutionary trajectories. This interpretation is consistent with the data of Hervé et al. (47), who showed that many mutated CLL antibodies regain auto-/polyreactive reactivity when reverted to their inferred germline configuration, indicating that somatic hypermutation often reshapes rather than creates *de novo* the functional properties of the leukemic BcR. In some subsets, this remodeling may attenuate the original autoreactivity in cells with features consistent with an anergic state (43, 48–50), as appears plausible for subset #4; in others, it may also contribute to the acquisition of autonomous signaling properties. Subset #2 may represent a more complex case, in which the induction of anergy may be less prominent or absent despite evidence of somatic mutation. This could reflect a particular developmental trajectory in which selection is driven mainly by more conventional antigenic stimuli, while the final leukemic configuration still depends on acquisition of specific structural features linked to self-recognition and autonomous signaling. This would be consistent with the more aggressive clinical features of subset #2 CLL clones (10, 51). However, it should be noted that in its final leukemic configuration subset #2 BcRs still display a certain degree of autoreactivity (36). Thus, healthy-donor repertoires do not point to a single common developmental route, but rather to multiple physiological trajectories emerging from a pre-existing repertoire that already contains CLL-compatible receptor configurations.

An additional complexity for CLL ontogeny is raised by the recurrent observation of oligoclonal or multiclonal expansions at both pre-leukemic and overt leukemic stages, indicating that multiple B-cell clones with CLL-like features persist and expand, rather than being fully eliminated or outcompeted from the outset. This is supported by the oligoclonality in low-count and CLL-type MBL (52) and by the identification of multiple productive clonally unrelated *IGHV* rearrangements in CLL patients (53) and, in established CLL, the presence of numerous additional clones besides the clinically relevant clone (54). In the latter setting, the proportion of CLS-IG was increased among the additional distinct clones identified within the circulating CD5+ B-cell fraction of CLL patients; whether these clones are truly non-leukemic or instead represent pre-leukemic expansions remains a hypothesis that warrants further evaluation.

In this sense, CLL development may proceed through at least three partially overlapping phases (depicted in **Figure 2**): (i) physiological generation of CLS-IG within the normal repertoire; (ii) emergence and persistence of oligoclonal or multiclonal expansions with CLL-like immunogenetic features; and (iii) progressive selection of one or a few dominant clone(s) under the combined influence of BcR structure and function, microenvironmental support, and genetic lesions. This scheme should be regarded as a conceptual framework rather than as an established or obligatory linear sequence. Moreover, individual clones may follow alternative routes, and some of the proposed phases may overlap or be bypassed.

**Figure 2:**
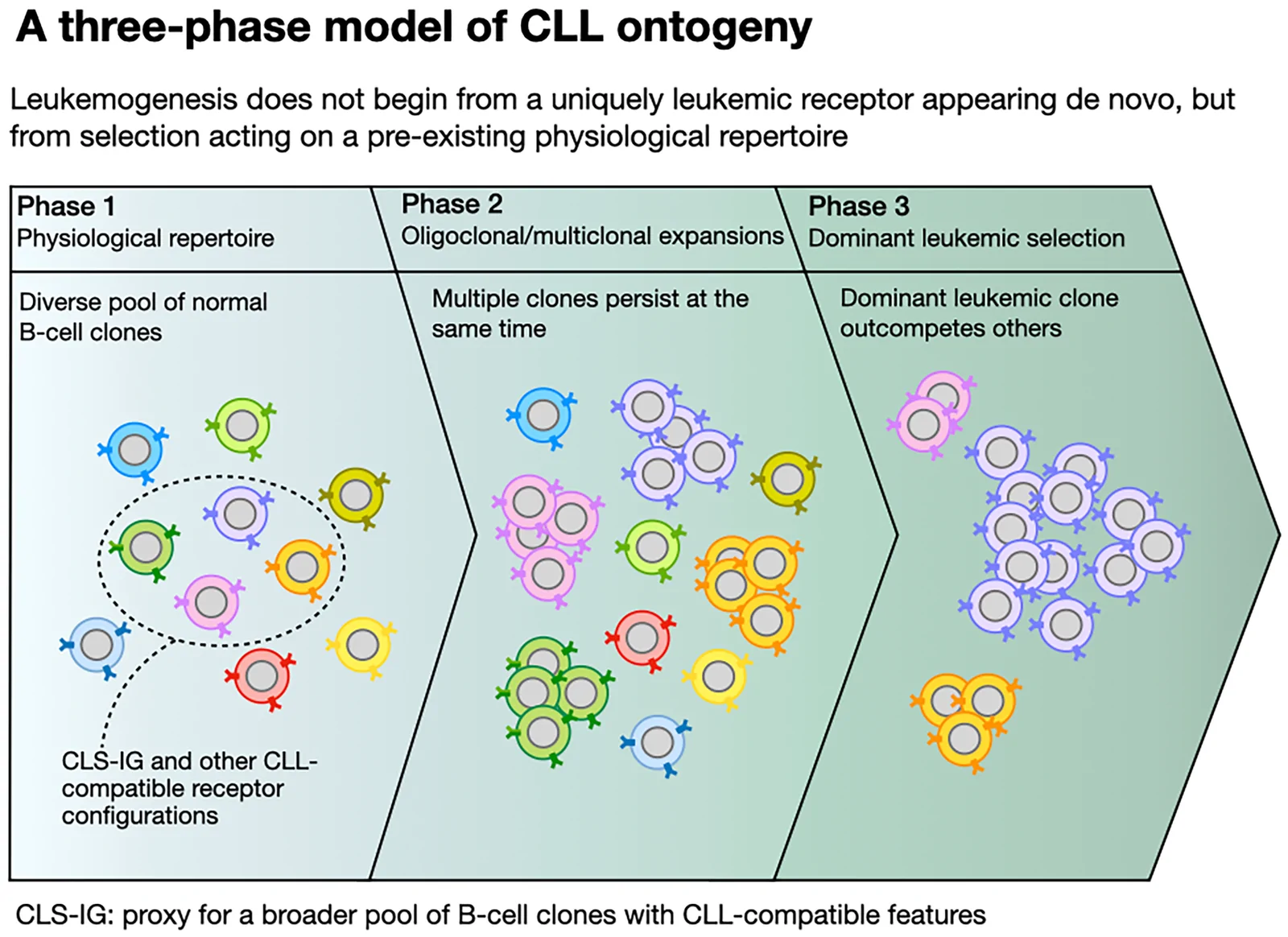
A three-phase model of CLL ontogeny. CLL development may proceed through at least three partially overlapping phases: (i) physiological generation of CLL-like stereotyped immunoglobulin rearrangements (CLS-IG) within the normal repertoire; (ii) emergence and persistence of oligoclonal or multiclonal expansions with CLL-like immunogenetic features; and (iii) progressive selection of one or a few dominant clones under the combined influence of B-cell receptor immunoglobulin structure and function, microenvironmental support, and genetic lesions. Importantly, this model is not restricted to rearrangements fulfilling formal CLS-IG criteria. In addition, the transitions depicted should be regarded as a conceptual model integrating healthy-repertoire observations with functional and genetic evidence from CLL, rather than as directly demonstrated lineage relationships.

Within this working model, the transition from phase II to phase III can be interpreted as a process of progressive clonal selection, whereas a key open question concerns how some physiologically generated clones enter a state of persistent oligoclonal or multiclonal expansion. One possible explanation is the emergence of a permissive state in which B-cell clones carrying CLL-compatible receptors are no longer fully constrained by tolerance and homeostatic control and can therefore persist and expand. This does not necessarily imply a complete failure of immune tolerance; rather, it may reflect relative permissiveness toward auto-/polyreactive BcR, chronic low-level antigenic stimulation, supportive microenvironmental signals, or early molecular lesions, possibly acquired in hematopoietic stem/progenitor cells or at pre-B-cell stages (55–58), that together establish a background favorable to the parallel persistence and expansion of multiple clones. In this framework, phase II would represent one possible intermediate state in which a subset of physiologically generated B-cell clones carrying CLL-compatible receptor features becomes biologically maintained and selectable, thereby contributing to the oligoclonal or multiclonal expansions observed in MBL and overt CLL. Phase II could also be seen as part of a more complex process of clonal hematopoiesis (56, 57) possibly unbalanced towards the production of B cells.

It should also be noted that formal proof is still lacking that non-leukemic CLS-IG display the same self-recognition and/or autonomous-signaling properties described for some leukemic stereotyped BcR.

Overall, the available evidence supports a model in which CLL does not originate from cells with unique leukemic receptors appearing *de novo*, but from a selection of cells with a broader physiological repertoire of convergent BcR configurations. The challenge ahead is therefore not only to define the antigen-binding and reactivity properties of these BcR, many of which are known to display auto-/polyreactivity and/or reactivity toward selected microbial elements (38, 39, 59–61), but also to understand the biological context that allows such clones to achieve persistent clonal expansion and leukemic transformation.

## References

[1] Fais F Ghiotto F Hashimoto S Sellars B Valetto A Allen SL . Chronic lymphocytic leukemia B cells express restricted sets of mutated and unmutated antigen receptors. J Clin Invest. (1998) 102:1515–25. doi: 10.1172/jci3009 9788964 PMC509001

[2] Schroeder HW Dighiero G . The pathogenesis of chronic lymphocytic leukemia: analysis of the antibody repertoire. Immunol Today. (1994) 15:288–94. doi: 10.1016/0167-5699(94)90009-4 7520700

[3] Messmer BT Albesiano E Efremov DG Ghiotto F Allen SL Kolitz J . Multiple distinct sets of stereotyped antigen receptors indicate a role for antigen in promoting chronic lymphocytic leukemia. J Exp Med. (2004) 200:519–25. doi: 10.1084/jem.20040544 15314077 PMC2211936

[4] Agathangelidis A Darzentas N Hadzidimitriou A Brochet X Murray F Yan X-J . Stereotyped B-cell receptors in one-third of chronic lymphocytic leukemia: a molecular classification with implications for targeted therapies. Blood. (2012) 119:4467–75. doi: 10.1182/blood-2011-11-393694 22415752 PMC3392073

[5] Stamatopoulos K Belessi C Moreno C Boudjograh M Guida G Smilevska T . Over 20% of patients with chronic lymphocytic leukemia carry stereotyped receptors: pathogenetic implications and clinical correlations. Blood. (2006) 108:26. doi: 10.1182/blood.v108.11.26.26 16985177

[6] Agathangelidis A ERIC the ERI on C on behalf of Chatzidimitriou A Gemenetzi K Giudicelli V Karypidou M . Higher-order connections between stereotyped subsets: implications for improved patient classification in CLL. Blood. (2021) 137:1365–76. doi: 10.1182/blood.2020007039 32992344 PMC7976441

[7] Widhopf GF Rassenti LZ Toy TL Gribben JG Wierda WG Kipps TJ . Chronic lymphocytic leukemia B cells of more than 1% of patients express virtually identical immunoglobulins. Blood. (2004) 104:2499–504. doi: 10.1182/blood-2004-03-0818 15217828

[8] Damle RN Wasil T Fais F Ghiotto F Valetto A Allen SL . Ig V gene mutation status and CD38 expression as novel prognostic indicators in chronic lymphocytic leukemia. Blood. (1999) 94:1840–7. doi: 10.1182/blood.v94.6.1840 10477712

[9] Hamblin TJ Davis Z Gardiner A Oscier DG Stevenson FK . Unmutated Ig V(H) genes are associated with a more aggressive form of chronic lymphocytic leukemia. Blood. (1999) 94:1848–54. doi: 10.1182/blood.V94.6.1848 10477713

[10] Rossi D Gaidano G . Biological and clinical significance of stereotyped B-cell receptors in chronic lymphocytic leukemia. Haematologica. (2010) 95:1992–5. doi: 10.3324/haematol.2010.033241 21123439 PMC2995555

[11] Baliakas P Hadzidimitriou A Sutton L-A Minga E Agathangelidis A Nichelatti M . Clinical effect of stereotyped B-cell receptor immunoglobulins in chronic lymphocytic leukaemia: a retrospective multicentre study. Lancet Haematology. (2014) 1:e74–84. doi: 10.1016/s2352-3026(14)00005-2 27030157

[12] Sutton L-A Young E Baliakas P Hadzidimitriou A Moysiadis T Plevova K . Different spectra of recurrent gene mutations in subsets of chronic lymphocytic leukemia harboring stereotyped B-cell receptors. Haematologica. (2016) 101:959–67. doi: 10.3324/haematol.2016.141812 27198719 PMC4967575

[13] Hacken E Gounari M Ghia P Burger JA . The importance of B cell receptor isotypes and stereotypes in chronic lymphocytic leukemia. Leukemia. (2018) 18:1–12. doi: 10.1038/s41375-018-0303-x 30555163 PMC7182338

[14] Gerousi M Laidou S Gemenetzi K Stamatopoulos K Chatzidimitriou A . Distinctive signaling profiles with distinct biological and clinical implications in aggressive CLL subsets with stereotyped B-cell receptor immunoglobulin. Front Oncol. (2021) 11:771454. doi: 10.3389/fonc.2021.771454 34804974 PMC8595110

[15] Tam C Thompson PA . BTK inhibitors in CLL: second-generation drugs and beyond. Blood Adv. (2024) 8:2300–9. doi: 10.1182/bloodadvances.2023012221 38478390 PMC11117011

[16] Forconi F Potter KN Sozzi E Henderson I Cencini E Rossi D . The IGHV1-69/IGHJ3 recombinations of unmutated CLL are distinct from those of normal B cells. Blood. (2012) 119:2106–9. doi: 10.1182/blood-2011-08-375501 22234701

[17] Forconi F Potter KN Wheatley I Darzentas N Sozzi E Stamatopoulos K . The normal IGHV1-69–derived B-cell repertoire contains stereotypic patterns characteristic of unmutated CLL. Blood. (2010) 115:71–7. doi: 10.1182/blood-2009-06-225813 19887677

[18] Seifert M Sellmann L Bloehdorn J Wein F Stilgenbauer S Dürig J . Cellular origin and pathophysiology of chronic lymphocytic leukemia. J Exp Med. (2012) 209:2183–98. doi: 10.1084/jem.20120833 23091163 PMC3501361

[19] Murphy WJ Collins CP Kohler H . The past, present, and future of anti-idiotype antibodies. Front Immunol. (2025) 16:1686107. doi: 10.3389/fimmu.2025.1686107 41333455 PMC12665528

[20] Briney B Inderbitzin A Joyce C Burton DR . Commonality despite exceptional diversity in the baseline human antibody repertoire. Nature. (2019) 566:393–7. doi: 10.1038/s41586-019-0879-y 30664748 PMC6411386

[21] Soto C Bombardi RG Branchizio A Kose N Matta P Sevy AM . High frequency of shared clonotypes in human B cell receptor repertoires. Nature. (2019) 23:1–18. doi: 10.1038/s41586-019-0934-8 30760926 PMC6949180

[22] Darzentas N Stamatopoulos K . Stereotyped B cell receptors in B cell leukemias and lymphomas. Methods Mol Biol (Clifton NJ). (2013) 971:135–48. doi: 10.1007/978-1-62703-269-8_8 23296962

[23] Ghiotto F Fais F Valetto A Albesiano E Hashimoto S Dono M . Remarkably similar antigen receptors among a subset of patients with chronic lymphocytic leukemia. J Clin Invest. (2004) 113:1008–16. doi: 10.1172/jci19399 15057307 PMC379317

[24] Tobin G Thunberg U Johnson A Eriksson I Söderberg O Karlsson K . Chronic lymphocytic leukemias utilizing the VH3-21 gene display highly restricted Vλ2-14 gene use and homologous CDR3s: implicating recognition of a common antigen epitope. Blood. (2003) 101:4952–7. doi: 10.1182/blood-2002-11-3485 12586612

[25] Marcatili P Ghiotto F Tenca C Chailyan A Mazzarello AN Yan X-J . Igs expressed by chronic lymphocytic leukemia B cells show limited binding-site structure variability. J Immunol. (2013) 190:5771–8. doi: 10.4049/jimmunol.1300321 23636053

[26] Bagnara D Colombo M Reverberi D Matis S Massara R Cardente N . Characterizing features of human circulating B cells carrying CLL-like stereotyped immunoglobulin rearrangements. Front Oncol. (2022) 12:894419. doi: 10.3389/fonc.2022.894419 35837088 PMC9275393

[27] Dupont M Derouault P Pascal V Dmytruk N Roubinet M Rivière C . CLL‐like stereotyped BCRs are equally produced in young and old healthy adults, share features of public clonotypes and may be stuck at a pre‐germinal centre stage. Br J Haematol. (2026) 208:106–15. doi: 10.1111/bjh.70204 41117149

[28] Muggen AF Jong M Wolvers-Tettero ILM Kallemeijn MJ Teodósio C Darzentas N . The presence of CLL-associated stereotypic B cell receptors in the normal BCR repertoire from healthy individuals increases with age. Immun Ageing. (2019) 16:1–12. doi: 10.1186/s12979-019-0163-x 31485252 PMC6714092

[29] Vergani S Bagnara D Agathangelidis A Ng AKY Ferrer G Mazzarello AN . CLL stereotyped B-cell receptor immunoglobulin sequences are recurrent in the B-cell repertoire of healthy individuals: apparent lack of central and early peripheral tolerance censoring. Front Oncol. (2023) 13:1112879. doi: 10.3389/fonc.2023.1112879 37007084 PMC10063922

[30] Colombo M Bagnara D Reverberi D Matis S Cardillo M Massara R . Tracing CLL-biased stereotyped immunoglobulin gene rearrangements in normal B cell subsets using a high- throughput immunogenetic approach. Mol Med. (2020) 26:1–10. doi: 10.1186/s10020-020-00151-9 32156260 PMC7063734

[31] Agathangelidis A Galigalidou C Scarfò L Moysiadis T Rovida A Gounari M . Infrequent “chronic lymphocytic leukemia-specific” immunoglobulin stereotypes in aged individuals with or without low-count monoclonal B-cell lymphocytosis. Haematologica. (2021) 106:1178–81. doi: 10.3324/haematol.2020.247908 32586905 PMC8018099

[32] Chiorazzi N Ferrarini M . Cellular origin(s) of chronic lymphocytic leukemia: cautionary notes and additional considerations and possibilities. Blood. (2011) 117:1781–91. doi: 10.1182/blood-2010-07-155663 21148333 PMC3056635

[33] Sethna Z Elhanati Y Callan CG Walczak AM Mora T . OLGA: fast computation of generation probabilities of B- and T-cell receptor amino acid sequences and motifs. Bioinformatics. (2019) 35:2974–81. doi: 10.1093/bioinformatics/btz035 30657870 PMC6735909

[34] Chu CC Catera R Hatzi K Yan X-J Zhang L Wang XB . Chronic lymphocytic leukemia antibodies with a common stereotypic rearrangement recognize nonmuscle myosin heavy chain IIA. Blood. (2008) 112:5122–9. doi: 10.1182/blood-2008-06-162024 18812466 PMC2597608

[35] Liu Y Padhorny D Catera R Nicolo A Yan X-J Li SX . Conventional and non-conventional antigen-binding sites promote the development and function of chronic lymphocytic leukemia stereotyped subset #4 clones. Front Immunol. (2025) 16:1607189. doi: 10.3389/fimmu.2025.1607189 40918146 PMC12408587

[36] Cocomazzi PG Iatrou A Minici C Gounari M Linder AT Akpınaroğlu C . Defective cell-autonomous signalling and antigenic polyreactivity of B-cell receptors from chronic lymphocytic leukaemia stereotyped subset 1. Nat Commun. (2025) 17:1280. doi: 10.1038/s41467-025-68040-5 41469389 PMC12868647

[37] Minden MD Übelhart R Schneider D Wossning T Bach MP Buchner M . Chronic lymphocytic leukaemia is driven by antigen-independent cell-autonomous signalling. Nature. (2012) 489:309–12. doi: 10.1038/nature11309 22885698

[38] Hatzi K Catera R Atanasio CM Fischetti VA Allen SL Kolitz JE . Chronic lymphocytic leukemia immunoglobulins display bacterial reactivity that converges and diverges from auto-/poly-reactivity and IGHV mutation status. Clin Immunol. (2016) 172:44–51. doi: 10.1016/j.clim.2016.08.020 27586592

[39] Hoogeboom R Kessel KPM Hochstenbach F Wormhoudt TA Reinten RJA Wagner K . A mutated B cell chronic lymphocytic leukemia subset that recognizes and responds to fungi. J Exp Med. (2013) 210:59–70. doi: 10.1084/jem.20121801 23296468 PMC3549718

[40] Minici C Gounari M Übelhart R Scarfò L Minden MD Schneider D . Distinct homotypic B-cell receptor interactions shape the outcome of chronic lymphocytic leukaemia. Nat Commun. (2017) 8:1–12. doi: 10.1038/ncomms15746 28598442 PMC5472768

[41] Bagnara D Mazzarello AN Colombo M Nano E Cardente N Ferrero F . Immunogenetic architecture of chronic lymphocytic leukemia at early stage: insights from the O-CLL1 cohort. Antibodies. (2026) 15:25. doi: 10.3390/antib15020025 41874030 PMC13010646

[42] Stevenson FK Stamatopoulos K . The singularity of IGHV4-34 links infection, autoimmunity, and B-cell tumors. Blood. (2026) 147:1793–803. doi: 10.1182/blood.2025032357 41557911

[43] Burnett DL Reed JH Christ D Goodnow CC . Clonal redemption and clonal anergy as mechanisms to balance B cell tolerance and immunity. Immunol Rev. (2019) 292:61–75. doi: 10.1111/imr.12808 31556969

[44] Ntoufa S Papakonstantinou N Apollonio B Gounari M Galigalidou C Fonte E . B cell anergy modulated by TLR1/2 and the miR-17∼92 cluster underlies the indolent clinical course of chronic lymphocytic leukemia stereotyped subset #4. J Immunol. (2016) 196:4410–7. doi: 10.4049/jimmunol.1502297 27059597

[45] Potter KN Hobby P Klijn S Stevenson FK Sutton BJ . Evidence for involvement of a hydrophobic patch in framework region 1 of human V4-34-encoded Igs in recognition of the red blood cell I antigen. J Immunol. (2002) 169:3777–82. doi: 10.4049/jimmunol.169.7.3777 12244172

[46] Kolijn PM Hosnijeh FS Späth F Hengeveld PJ Agathangelidis A Saleh M . High-risk subtypes of chronic lymphocytic leukemia are detectable as early as 16 years prior to diagnosis. Blood. (2022) 139:1557–63. doi: 10.1182/blood.2021012890 34662377 PMC10650964

[47] Hervé M Xu K Ng Y-S Wardemann H Albesiano E Messmer BT . Unmutated and mutated chronic lymphocytic leukemias derive from self-reactive B cell precursors despite expressing different antibody reactivity. J Clin Invest. (2005) 115:1636–43. doi: 10.1172/jci24387 15902303 PMC1088018

[48] Muzio M Apollonio B Scielzo C Frenquelli M Vandoni I Boussiotis V . Constitutive activation of distinct BCR-signaling pathways in a subset of CLL patients: a molecular signature of anergy. Blood. (2008) 112:188–95. doi: 10.1182/blood-2007-09-111344 18292287

[49] Packham G Krysov S Allen A Savelyeva N Steele AJ Forconi F . The outcome of B-cell receptor signaling in chronic lymphocytic leukemia: proliferation or anergy. Haematologica. (2014) 99:1138–48. doi: 10.3324/haematol.2013.098384 24986876 PMC4077074

[50] Dizon BLP Holla P Mutic EC Schaughency P Pierce SK . Human naïve B cells show evidence of anergy and clonal redemption following vaccination. NPJ Vaccines. (2025) 10:96. doi: 10.1038/s41541-025-01133-w 40368926 PMC12078529

[51] Baliakas P Agathangelidis A Hadzidimitriou A Sutton L-A Minga E Tsanousa A . Not all IGHV3-21 chronic lymphocytic leukemias are equal: prognostic considerations. Blood. (2015) 125:856–9. doi: 10.1182/blood-2014-09-600874 25634617 PMC4311230

[52] Agathangelidis A Iatrou A Pechlivanis N Karakatsoulis G Zaragoza-Infante L Galigalidou C . Molecular and functional characterization of the B-cell receptor in chronic lymphocytic leukemia-like monoclonal B-cell lymphocytosis. Leukemia. (2026) 40:454–8. doi: 10.1038/s41375-025-02797-y 41501505 PMC12875869

[53] Stamatopoulos B Timbs A Bruce D Smith T Clifford R Robbe P . Targeted deep sequencing reveals clinically relevant subclonal IgHV rearrangements in chronic lymphocytic leukemia. Leukemia. (2017) 31:837–45. doi: 10.1038/leu.2016.307 27795555

[54] Bagnara D Mazzarello AN Cardente N Vergani S Kasar S Fernandes S . Chronic lymphocytic leukemia often arises by a multiclonal selection process. Haematologica. (2025) 110:1747–57. doi: 10.3324/haematol.2024.286380 39973373 PMC12358785

[55] Chiang C-L Hu EY Chang L Labanowska J Zapolnik K Mo X . Leukemia-initiating HSCs in chronic lymphocytic leukemia reveal clonal leukemogenesis and differential drug sensitivity. Cell Rep. (2022) 40:111115. doi: 10.1016/j.celrep.2022.111115 35858552

[56] Al-Sawaf O Locher BN Christen F Hablesreiter R Rajamani A Robrecht S . The landscape and evolution of clonal hematopoiesis in chronic lymphocytic leukemia∗. Blood. (2026) 147:1323–37. doi: 10.1182/blood.2025029905 41528026 PMC13014086

[57] Martino EA Caserta S Skafi M Alvaro ME Bruzzese A Amodio N . Clonal haematopoiesis in chronic lymphocytic leukaemia: Biology, inflammaging and clinical implications in the era of targeted therapy. Clin Transl Med. (2026) 16:e70633. doi: 10.1002/ctm2.70633 41793184 PMC12966884

[58] Condoluci A Rossi D . Age-related clonal hematopoiesis and monoclonal B-cell lymphocytosis/chronic lymphocytic leukemia: a new association? Haematologica. (2018) 103:751–2. doi: 10.3324/haematol.2018.191098 29712819 PMC5927972

[59] Gounari M Ntoufa S Apollonio B Papakonstantinou N Ponzoni M Chu CC . Excessive antigen reactivity may underlie the clinical aggressiveness of chronic lymphocytic leukemia stereotyped subset #8. Blood. (2015) 125:3580–7. doi: 10.1182/blood-2014-09-603217 25900981 PMC4458798

[60] Chu CC Catera R Zhang L Didier S Agagnina BM Damle RN . Many chronic lymphocytic leukemia antibodies recognize apoptotic cells with exposed nonmuscle myosin heavy chain IIA: Implications for patient outcome and cell of origin. Blood. (2010) 115:3907–15. doi: 10.1182/blood-2009-09-244251 20110421 PMC2869555

[61] Catera R Silverman GJ Hatzi K Seiler T Didier S Zhang L . Chronic lymphocytic leukemia cells recognize conserved epitopes associated with apoptosis and oxidation. Mol Med. (2008) 14:665–74. doi: 10.2119/2008-00102.catera 19009014 PMC2582860

